# Supplementary material for: teiresias, a Fruitless target gene encoding an immunoglobulin-superfamily transmembrane protein, is required for neuronal feminization in Drosophila
Source: Commun Biol. 2020 Oct 21;3:598. doi: 10.1038/s42003-020-01327-z (PMC7578032; doi:10.1038/s42003-020-01327-z)
Supplement: Supplementary file 1 — Supplementary Information [file 42003_2020_1327_MOESM1_ESM.pdf]

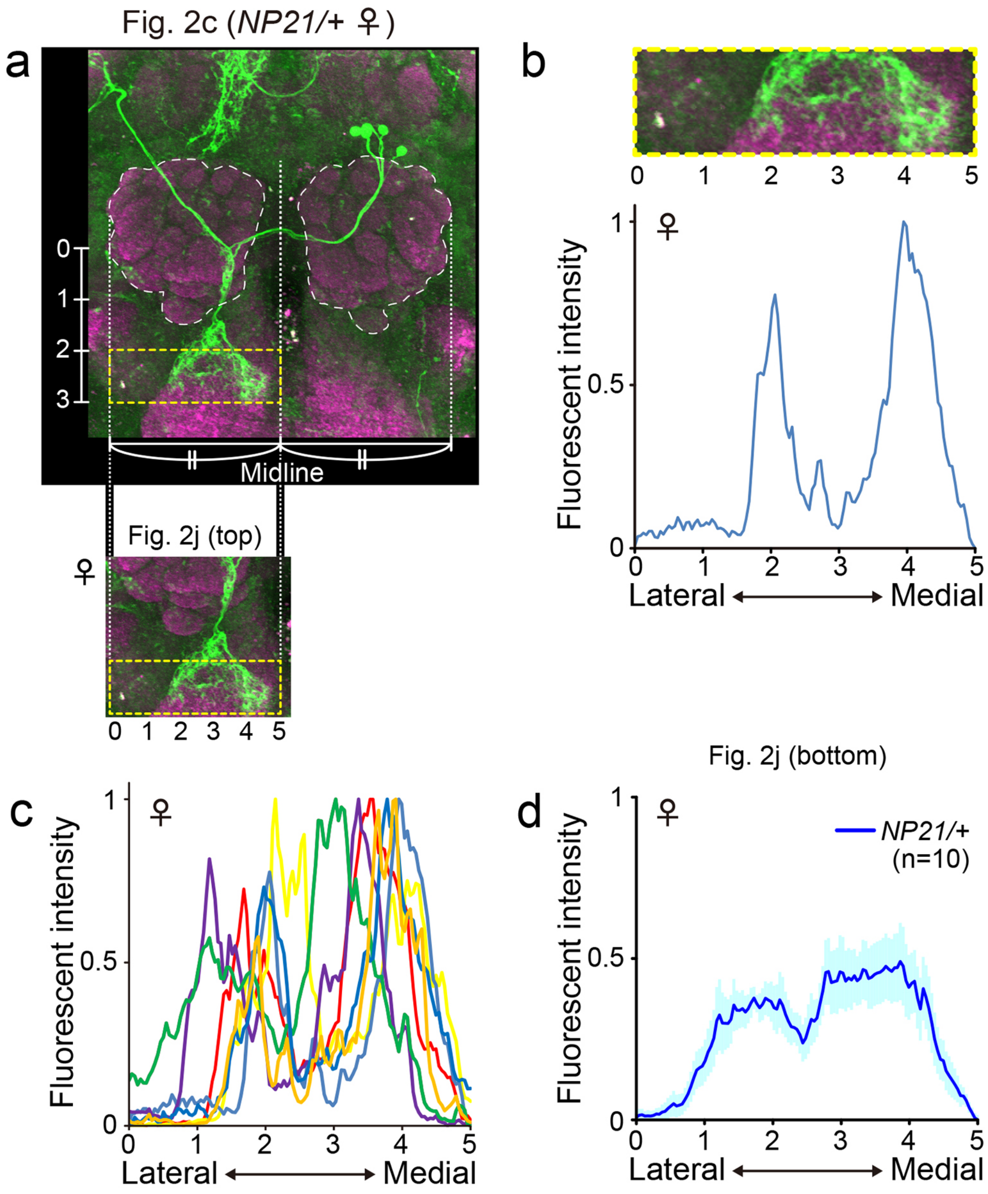

Supplementary Fig. 1 Arboplot analysis of mAL neurons.

(**a**,**b**) The method to quantify the fluorescence distribution in single neural images. To scale the mAL neurites, we first determine the left and right ends of the paired antennal lobe in a given specimen (the contour of antennal lobes is highlighted by white broken lines), and the middle point of the two ends defines the midline. We then draw a square, a side of which coincides with the half distance from the midline to a lateral edge of the antennal lobe on the neural image; the square is positioned so that its one vertical side superimposes on the midline while the upper side passes through the branching point at which the contralateral neurite bifurcates into the ascending and descending components (upper panel). The vertical (rostral) side of this square defines the y-axis (0-3) whereas the horizontal (lateral) side defines the x-axis (0-5 for a hemisphere) of the neural image subjected to fluorescence intensity measurements. We measure the fluorescent intensity of all pixels within the bottom one third (boxed with a yellow line) and the integrated intensity (0-1) for every x-axis point is plotted in the central to lateral direction as shown in the lower panel (arboplot). (**c**,**d**) Fluorescence intensity quantification with multiple neural images. Examples of the arboplots for ten different brains (**c**) and an average arboplot for the illustrated ten traces (**d**).

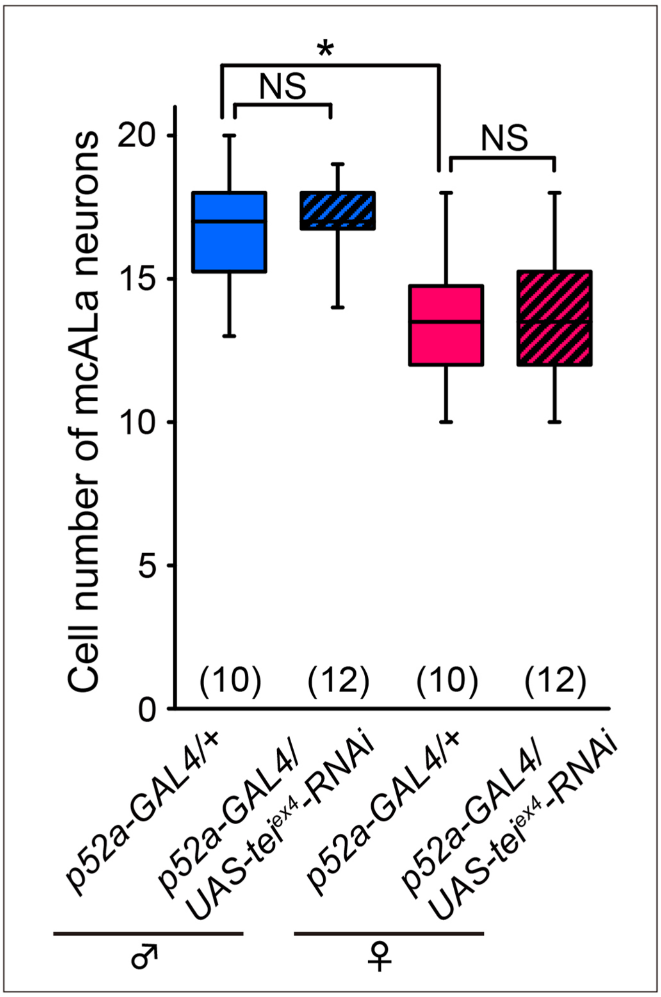

Supplementary Fig. 2 The number of cells composing the mcALa cluster.
tei knockdown does not affect the number of cells in the mcALa cluster in males and females. The number of flies examined is shown in parentheses. The box-and-whisker plot shows the first quartile (25th percentile), median, third quartile (75th percentile) and minimum and maximum of each set of data. The statistical significance of differences was evaluated by Kruskal-Wallis analysis followed by Steel-Dwass nonparametric multiple comparisons: *P<0.05; NS, not significant.

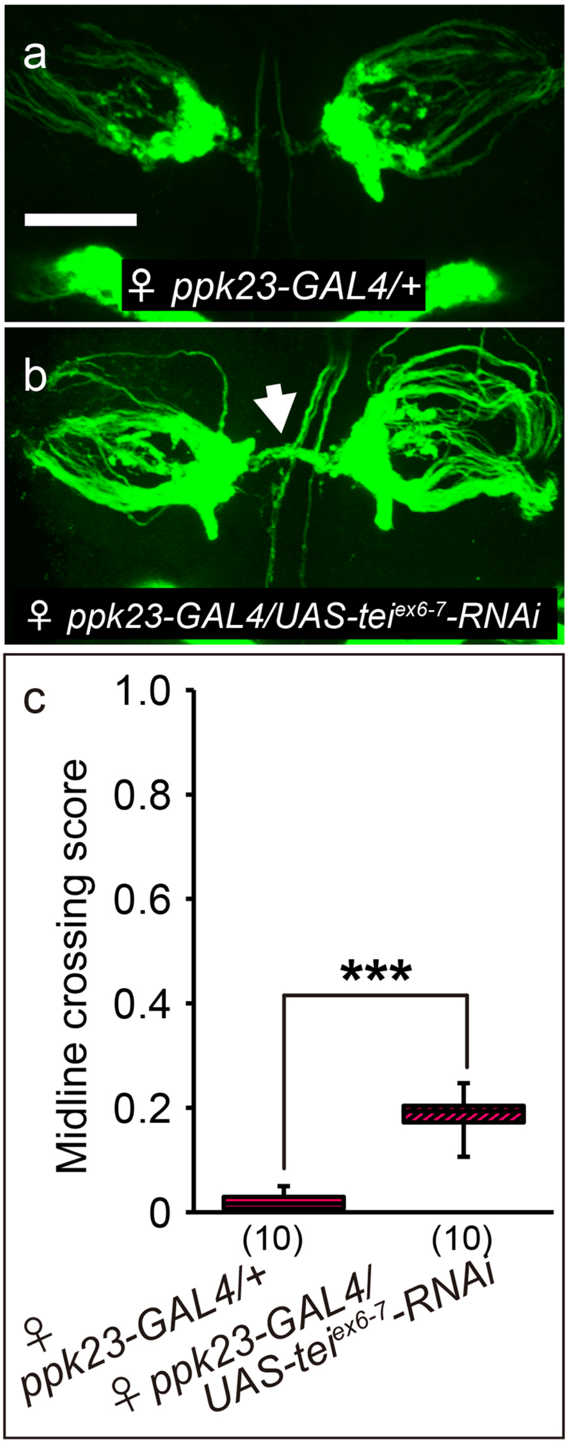

Supplementary Fig. 3 ***tei*** **knockdown with *UAS-tei^{ex6-7}-RNAi* increases midline crossing of *ppk-GAL4*-positive sensory axons in female flies.**

a A control female. **b** A female with *tei* knockdown has midline crossing fibers (arrow). **c** Quantitative comparisons of midline crossing scores. Statistical differences were evaluated by Mann-Whitney U-test: ***P<0.001. The scale bar represents 50 μm.

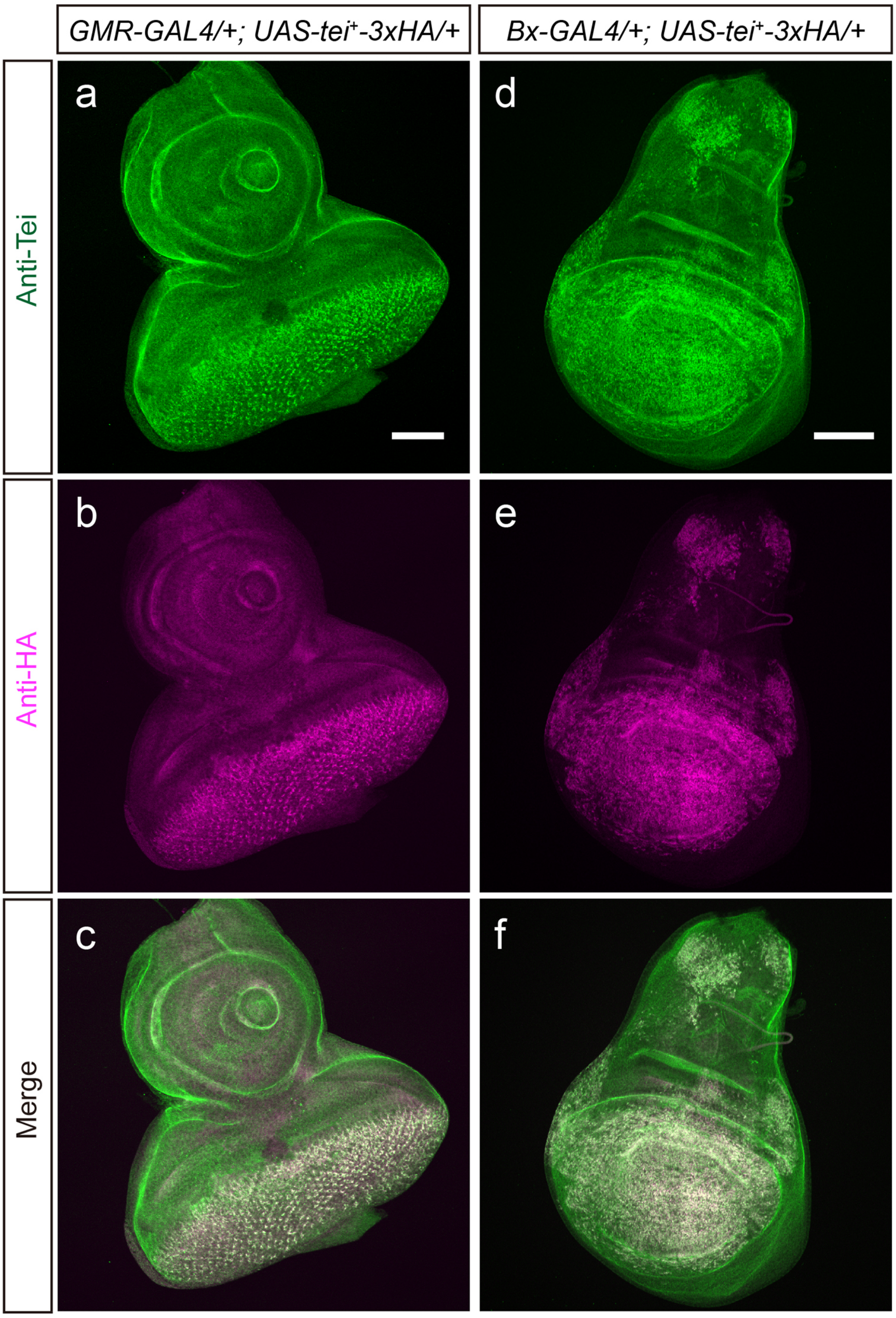

Supplementary Fig. 4 Newly raised anti-Tei antibody recognizes overexpressed Tei with the HA epitope-tag. *GMR-GAL4* and *Bx-GAL4* were used to drive *UAS-tei^{+}-3xHA* in the eye-antennal disc (**a-c**) and wing disc (**d-f**), respectively. Double staining of tissues with the anti-Tei (green) and anti-HA (magenta) antibodies revealed that these two antibodies label exactly the same cells according to the known expression patterns of the respective GAL4 drivers. Scale bar: 50 μm.

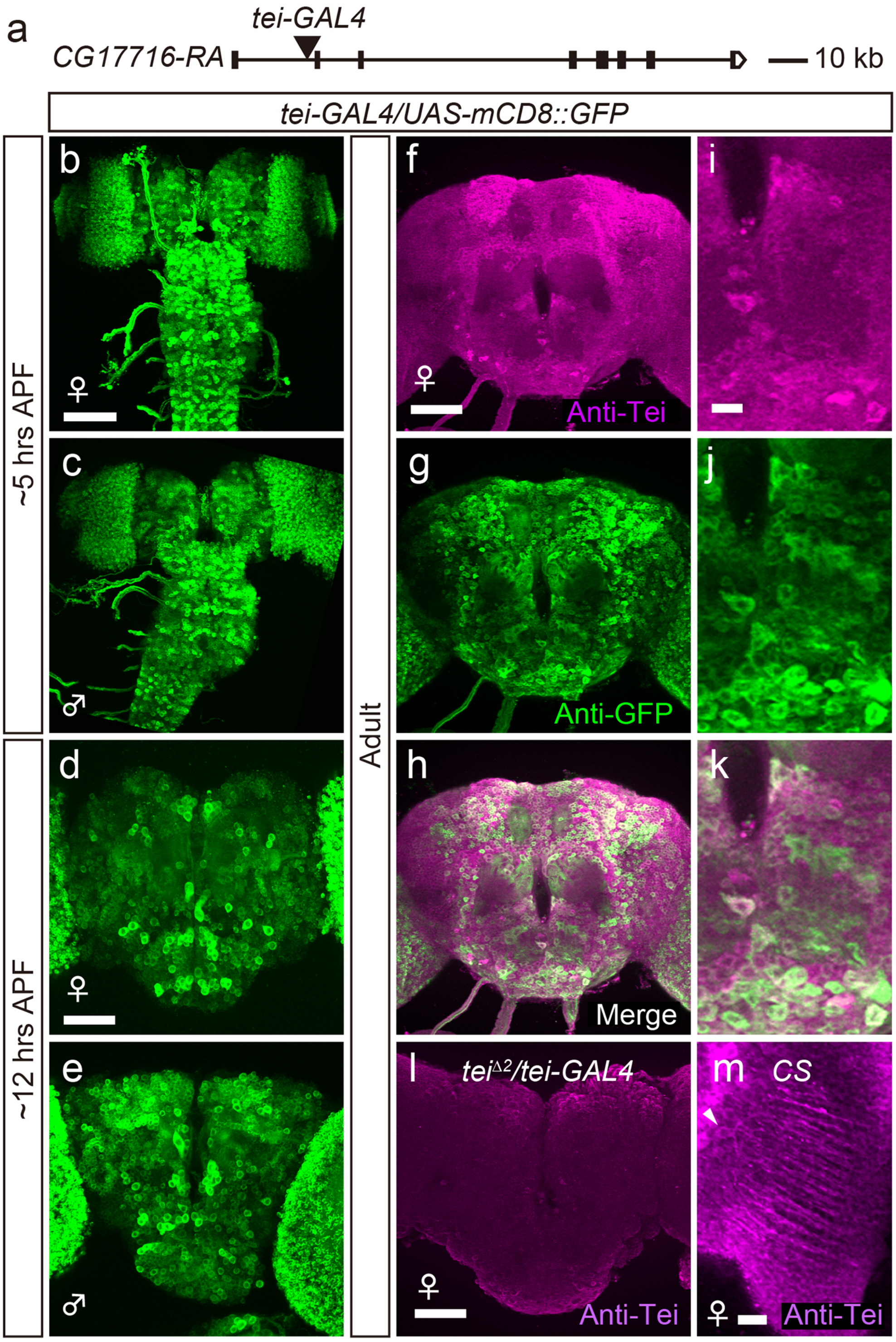

Supplementary Fig. 5 ***tei*** **expression in the CNS.**

a Localization of *tei-GAL4* in the *tei* locus. **b-e** *tei-GAL4* expression in the entire CNS at ~5 h APF (**b**, **c**) or in the brain at ~12 h APF (**d**, **e**) dissected from a female (**b**, **d**) or male (**c**, **e**) pupa. (**f-k**) The brain cells immunopositive for the anti-Tei antibody (**f**, **h**; magenta) coincide with those expressing the *tei-GAL4* reporter (**g**, **h**; green) in the adult. High magnification images are shown in **i-k**. **l** Anti-Tei immunoreactivity diminishes in the *tei* mutant brain. **m** A magnified view of the neurites of a few optic lobe neurons from a CS female stained with the anti-Tei antibody. The brain images shown are z-projections. Scale bars represent 50 µm (**b**, **d**, **f**, **l**) and 10 µm (**i**, **m**).

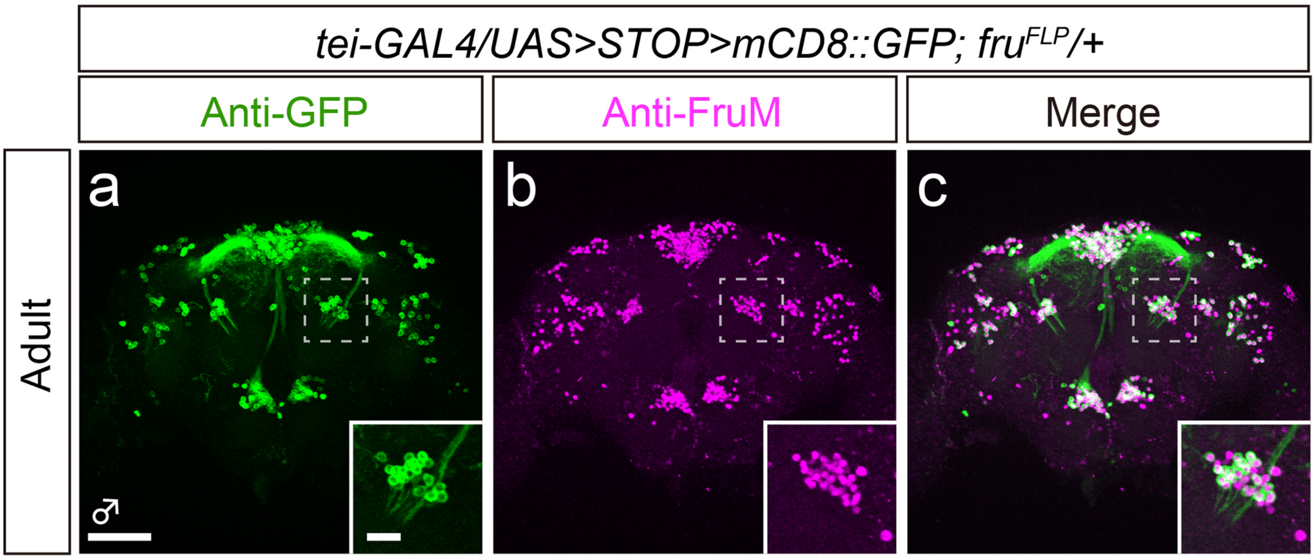

Supplementary Fig. 6 All *fru*^{FLP} neurons are also positive for *tei-GAL4* in the adult male brain.
The tissue was double stained with the anti-GFP antibody (**a**) and anti-FruM antibody (**b**). A merged image is shown in (**c**). The region boxed with a white broken line is shown in an inset at a higher magnification. A male-adult brain was double-stained with the anti-GFP and anti-FruM antibodies in an immunostaining-enhancing solution (Immuno-shot; Cosmo Bio, IS-F-20); the fly used for staining carried an intersection triad: *tei-GAL4*, *fru*^{FLP} and *UAS>STOP>mCD8::GFP* (highlighted by the box drawn with a white broken line is an mAL cluster that contained 18 Tei and FruM doubly positive neurons). Scale bars: 50 μm and 10 μm (for insets).

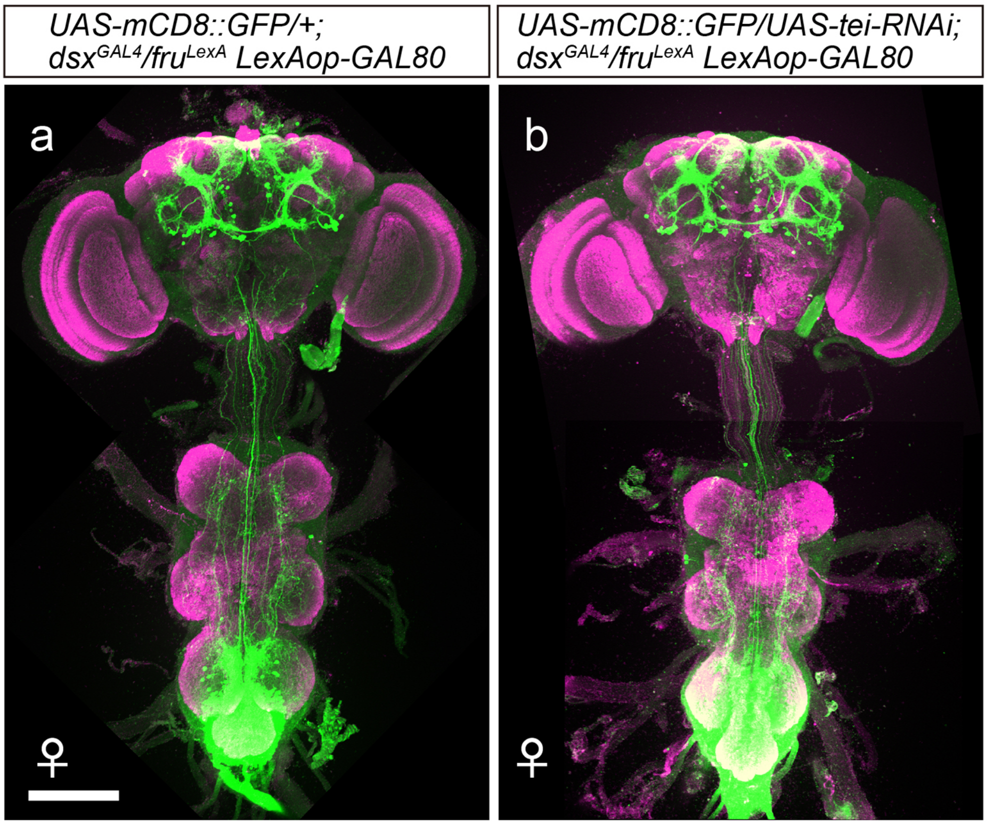

Supplementary Fig. 7 ***tei*** **knockdown in the** ***dsx*****-positive CNS neurons.**

GAL80 expression confined to *fru*-positive cells allowed *tei* knockdown restricted to *dsx*-positive cells that do not express *fru* in female flies. Scale bar: 100 μm.

Supplementary Table 1. List of primers used in this study.

#	Name	Sequence 5'-3'	note
1	Tei F	TCGATAAGGCGTGAACATTCCGG	qPCR primer (tei)
2	Tei R	CTGCCACGGAAACATCCTGATCA	qPCR primer (tei)
3	RpL32 (rp49) F	AGATCGTGAAGAAGCGCACCAAG	qPCR primer (rp49)
4	RpL32 (rp49) R	CACCAGGAACTTCTTGAATCCGG	qPCR primer (rp49)
5	Tei-F1	CGTGGATTATTGACCCGTTT	tei-GAL4
6	GAL4 R156	GCTTGGTCTTGGGGCTGTAG	tei-GAL4
7	Tei-ex4 RNAi top	ctagcagtCATTCTTGTACCACATGCTGGtagttatattcaagcataCCAGCATGTGGTACAAGAATGgcg	UAS-tei-ex4-RNAi
8	Tei-ex4 RNAi bottom	aattcgcCCAGCATGTGGTACAAGAATGtatgcttgaatataactaCATTCTTGTACCACATGCTGGactg	UAS-tei-ex4-RNAi
9	Tei-ex6 RNAi top	ctagcagtGGTAGTGGTTGCGGATGTGCGtagttatattcaagcataCGCACATCCGCAACCACTACCgcg	UAS-tei-ex6-7-RNAi
10	Tei-ex6 RNAi bottom	aattcgcCGCACATCCGCAACCACTACCtatgcttgaatataactaGGTAGTGGTTGCGGATGTGCGactg	UAS-tei-ex6-7-RNAi
11	Tei-HA F	taaGAATTCaacATGCGCCCTACATTTGATCGCCG	UAS-tei+-3xHA
12	Tei-HA R	caaGGTACCctaAGCGTAATCTGGAACGTCATATGGATAGGATCCTGCATAGTCCGGGACGTCATAGGGATAGCCCGCATAGTCAGGAACATCGTATGGGTAatgtcggttcgtggtggcgaca	UAS-tei+-3xHA

F: forward primer, R: reverse primer

Supplementary Table 2. Genotypes of flies used in this study.

**Fig. 1**	
c	*elav-GAL4^{C155}/+*
	elav-GAL4^{C155}/+; UAS-fruBM^{+}/+
**Fig. 2**	
a, e	*yw hs-FLP/Y; FRTG13 tubP-GAL80/FRTG13 UAS-mCD8::GFP; NP21/+*
c, g	*yw hs-FLP/+; FRTG13 tubP-GAL80/FRTG13 UAS-mCD8::GFP; NP21/+*
b, f	*yw hs-FLP/Y; FRTG13 tubP-GAL80/FRTG13 UAS-mCD8::GFP; NP21/UAS-tei^{ex6-7}-RNAi*
d, h	*yw hs-FLP/+; FRTG13 tubP-GAL80/FRTG13 UAS-mCD8::GFP; NP21/UAS-tei^{ex6-7}-RNAi*
i-k	*yw hs-FLP/Y; FRTG13 tubP-GAL80/FRTG13 UAS-mCD8::GFP; NP21/+*
	yw hs-FLP/+; FRTG13 tubP-GAL80/FRTG13 UAS-mCD8::GFP; NP21/+
	yw hs-FLP/Y; FRTG13 tubP-GAL80/FRTG13 UAS-mCD8::GFP; NP21/UAS-tei^{ex6-7}-RNAi
	yw hs-FLP/+; FRTG13 tubP-GAL80/FRTG13 UAS-mCD8::GFP; NP21/UAS-tei^{ex6-7}-RNAi
l	*UAS-tei^{ex6-7}-RNAi/+*
	tei-GAL4/+; UAS-tei^{ex6-7}-RNAi/+
**Fig. 3**	
a, e	*yw hs-FLP/Y; FRTG13 tubP-GAL80/FRTG13 UAS-mCD8::GFP; NP21/+*
c, g	*yw hs-FLP/+; FRTG13 tubP-GAL80/FRTG13 UAS-mCD8::GFP; NP21/+*
b, f	*yw hs-FLP/Y; FRTG13 tubP-GAL80/FRTG13 UAS-mCD8::GFP; NP21/UAS-tei^{ex6-7}-RNAi*
d, h	*yw hs-FLP/+; FRTG13 tubP-GAL80/FRTG13 UAS-mCD8::GFP; NP21/UAS-tei^{ex6-7}-RNAi*
i	*yw hs-FLP/Y; FRTG13 tubP-GAL80/FRTG13 UAS-mCD8::GFP; NP21/+*
	yw hs-FLP/Y; FRTG13 tubP-GAL80/FRTG13 UAS-mCD8::GFP; NP21/UAS-tei^{ex6-7}-RNAi
j	*yw hs-FLP/+; FRTG13 tubP-GAL80/FRTG13 UAS-mCD8::GFP; NP21/+*

	yw hs-FLP/+; FRTG13 tubP-GAL80/FRTG13 UAS-mCD8::GFP; NP21/UAS-tei$^{ex6\text{-}7}$-RNAi
k	*yw hs-FLP/Y; FRTG13 tubP-GAL80/FRTG13 UAS-mCD8::GFP; NP21/+*
	yw hs-FLP/Y; FRTG13 tubP-GAL80/FRTG13 UAS-mCD8::GFP; NP21/UAS-tei$^{ex6\text{-}7}$-RNAi
	yw hs-FLP/+; FRTG13 tubP-GAL80/FRTG13 UAS-mCD8::GFP; NP21/+
	yw hs-FLP/+; FRTG13 tubP-GAL80/FRTG13 UAS-mCD8::GFP; NP21/UAS-tei$^{ex6\text{-}7}$-RNAi
**Fig. 4**	
a, c	*w/Y; UAS-mCD8::GFP/+; ppk23-GAL4/+*
b, d	*w/+; UAS-mCD8::GFP/+; ppk23-GAL4/+*
e	*w/Y; UAS-mCD8::GFP/UAS-tei^{ex4}-RNAi; ppk23-GAL4/+*
f	*w/+; UAS-mCD8::GFP/UAS-tei^{ex4}-RNAi; ppk23-GAL4/+*
h	*w/Y; UAS-mCD8::GFP/+; ppk23-GAL4/+*
i	*w/Y; UAS-mCD8::GFP/+; ppk23-GAL4/+*
	w/Y; UAS-mCD8::GFP/UAS-tei^{ex4}-RNAi; ppk23-GAL4/+
	w/+; UAS-mCD8::GFP/+; ppk23-GAL4/+
	w/+; UAS-mCD8::GFP/UAS-tei^{ex4}-RNAi; ppk23-GAL4/+
j	*w/Y; UAS-mCD8::GFP/+; ppk23-GAL4/+*
k	*w/Y; UAS-mCD8::GFP/+; ppk23-GAL4/UAS-tei^{+}-3xHA*
l	*w/Y; UAS-mCD8::GFP/+; ppk23-GAL4/+*
	w/Y; UAS-mCD8::GFP/+; ppk23-GAL4/UAS-tei^{+}-3xHA
m	*w/Y; FRTG13 UAS-mCD8::GFP/+; fru^{sat}, poxn-GAL4/fru^{2}*
n	*w/Y; FRTG13 UAS-mCD8::GFP/UAS-tei^{ex4}-RNAi; fru^{sat}, poxn-GAL4/fru^{2}*
o	*w/Y; FRTG13 UAS-mCD8::GFP/UAS-tei^{+}; fru^{sat}, poxn-GAL4/fru^{2}*
p	*w/Y; FRTG13 UAS-mCD8::GFP/+; fru^{sat}, poxn-GAL4/fru^{2}*
	w/Y; FRTG13 UAS-mCD8::GFP/UAS-tei^{ex4}-RNAi; fru^{sat}, poxn-GAL4/fru^{2}
	w/Y; FRTG13 UAS-mCD8::GFP/UAS-tei^{+}; fru^{sat}, poxn-GAL4/fru^{2}
q	*w/+; UAS-mCD8::GFP/UAS-robo1-RNAi; ppk23-GAL4/+*
r	*w/+; UAS-mCD8::GFP/UAS-robo1-RNAi; ppk23-GAL4/UAS-tei^{+}-3xHA*
s	*w/+; UAS-mCD8::GFP/UAS-robo1-RNAi; ppk23-GAL4/+*
	w/+; UAS-mCD8::GFP/UAS-robo1-RNAi; ppk23-GAL4/UAS-tei^{+}-3xHA

t	*w/+; UAS-mCD8::GFP/UAS-tei*^{ex4}*-RNAi; ppk23-GAL4/+*
u	*w/+; UAS-mCD8::GFP/+; ppk23-GAL4/UAS-robo1-RNAi*
v	*w/+; UAS-mCD8::GFP/UAS-tei*^{ex4}*-RNAi; ppk23-GAL4/UAS-robo1-RNAi*
w	*w/+; UAS-mCD8::GFP/UAS-tei*^{ex4}*-RNAi; ppk23-GAL4/+*
	w/+; UAS-mCD8::GFP/+; ppk23-GAL4/UAS-robo1-RNAi
	w/+; UAS-mCD8::GFP/UAS-tei^{ex4}*-RNAi; ppk23-GAL4/UAS-robo1-RNAi*
**Fig. 5**	
a-c	*w/+; tei-GAL4/UAS>STOP>mCD8::GFP; fru*^{FLP}*/+*
d-f	*w/Y; tei-GAL4/UAS>STOP>mCD8::GFP; fru*^{FLP}*/+*
g-i	*w/+; tei-GAL4/UAS>STOP>mCD8::GFP; fru*^{FLP}*/+*
j-l	*w/Y; tei-GAL4/UAS>STOP>mCD8::GFP; fru*^{FLP}*/+*
**Fig. 6**	
a-c	*w/+; tei-GAL4/UAS-mCD8::GFP*
d-f	*w/+; tei-GAL4/UAS-mCD8::GFP; UAS-fruBM/+*
g, h	*+/Y; Canton-S*
	+/Y; fru^{NP21}*/fru*^{2}
	+/Y; UAS-tei^{+}*/+; fru*^{NP21}*/fru*^{2}
	+/Y; fru^{GAL4}*/+*
	+/Y; UAS-tei^{+}*/+; fru*^{GAL4}*/+*
**Supplementary Fig. 1**	
a-d	*yw hs-FLP/+; FRTG13 tubP-GAL80/FRTG13 UAS-mCD8::GFP; NP21/+*
**Supplementary Fig. 2**	
	w/Y; p52a-GAL4/+
	w/Y; p52a-GAL4/UAS-tei^{ex4}*-RNAi*
	w/+; p52a-GAL4/+
	w/+; p52a-GAL4/UAS-tei^{ex4}*-RNAi*
**Supplementary Fig. 3**	
a	*w/+; UAS-mCD8::GFP/+; ppk23-GAL4/+*
b	*w/+; UAS-mCD8::GFP/+; ppk23-GAL4/UAS-tei*$^{ex6-7}$*-RNAi*
c	*w/+; UAS-mCD8::GFP/+; ppk23-GAL4/+*
	w/+; UAS-mCD8::GFP/+; ppk23-GAL4/UAS-tei^{ex6-7}*-RNAi*
**Supplementary Fig. 4**	

a-c	*w/+; GMR-GAL4/+; UAS-tei*$^{+}$*-3xHA/+*
d-f	*Bx-GAL4/+; UAS- tei*$^{+}$*-3xHA/+*
**Supplementary Fig. 5**	
b, d	*w/+; tei-GAL4/UAS-mCD8::GFP*
c, e	*w/Y; tei-GAL4/UAS-mCD8::GFP*
f-k	*w/+; tei-GAL4/UAS-mCD8::GFP*
l	*w/+; tei*$^{\Delta 2}$*/tei-GAL4*
m	*Canton-S*
**Supplementary Fig. 6**	
a-c	*w/Y; tei-GAL4/UAS>STOP>mCD8::GFP; fru*^{FLP}*/+*
**Supplementary Fig. 7**	
a	*w/+; UAS-mCD8::GFP/+; dsx*^{GAL4}*/fru*^{LexA} *LexAop-GAL80*
b	*w/+; UAS-mCD8::GFP/UAS-tei*^{ex4}*-RNAi; dsx*^{GAL4}*/fru*^{LexA} *LexAop-GAL80*
