# Supplementary material for: teiresias, a Fruitless target gene encoding an immunoglobulin-superfamily transmembrane protein, is required for neuronal feminization in Drosophila
Source: Commun Biol. 2020 Oct 21;3:598. doi: 10.1038/s42003-020-01327-z (PMC7578032; doi:10.1038/s42003-020-01327-z)
Supplement: Supplementary file 3 — Description of Additional Supplementary Files [file 42003_2020_1327_MOESM3_ESM.pdf]

Description of additional supplementary items

SUPPLEMENTARY DATA FILE LEGENDS

Supplementary Data 1. A list of query nucleotide sequences and the results of *in silico* similarity searches for genes with the Pal1 sequence.

Supplementary Data 2. Raw data used to construct graphs of figures shown in this paper.
